# Skip metastasis in papillary thyroid carcinoma is difficult to predict in clinical practice

**DOI:** 10.1186/s12885-017-3698-2

**Published:** 2017-10-25

**Authors:** Xilin Nie, Zhou Tan, Minghua Ge

**Affiliations:** Department of Head and Neck Surgery, Zhejiang Cancer Hospital, No.1 East Banshan Road, Hangzhou, 310022 People’s Republic of China

**Keywords:** Papillary thyroid carcinoma, Skip metastasis, Lateral lymph node metastasis, Central lymph node metastasis, Risk factors

## Abstract

**Background:**

Cervical lymph node metastases are very common in papillary thyroid cancer (PTC), and typically spread in a predictable stepwise fashion in clinical practice. However, lateral lymph node metastasis (LLNM) without central lymph node metastasis (CLNM) as skip metastasis is not rare in PTC. The aim of this study was to investigate the incidence, risk factors and pattern of skip metastasis in PTC.

**Methods:**

A total of 271 patients with PTC and suspicious LLN diagnosed by pre-operation examinations who underwent total thyroidectomy and central lymph node dissection plus lateral lymph node dissection between January 2008 and December 2011 were enrolled in this study. Clinicopathological features were collected, and the pattern of cervical lymph node metastasis and skip metastasis were analyzed.

**Results:**

The LLNM rate was 74.9% (203/271, diagnosed by postoperative pathology examination) and significantly associated with extrathyroid extension (ETE), primary tumor located at the upper pole, and CLNM (*p* < 0.05). The skip metastasis rate was 14.8% (30/203) and was more frequently found in microcarcinoma patients, especially when the primary tumor size was ≤0.5 cm (*p* = 0.001 OR = 12.9). However, skip metastasis was unrelated to the remaining factors examined.

**Conclusion:**

Small cancers with a pre-operation diagnosis of LLNM are more likely to have skip metastases, especially when the primary tumor size is less than 0.5 cm in diameter; however, this type of metastasis appears to develop in a random fashion. Thus, additional research is needed to identify potential predictive factors, such as a primary tumor located at the upper pole.

## Background

Papillary thyroid carcinoma (PTC) accounts for approximately 80.0% of all thyroid malignancies and generally grows slowly. Thus, as an indolent disease, the prognosis is good for the majority of patients [1, 2]. However, cervical lymph node metastasis very common in PTC and is associated with an increased risk of local regional recurrence and overall mortality in select patient populations, although it does not show a major effect on prognosis [3–5]. As a result, controlling loco-regional recurrence has become a major challenge for most thyroid surgeons [6].

Many previous studies have reported that the dissemination of PTC cells through the lymphatic system occurs in a largely predictable stepwise fashion [7, 8]. Lymph node metastasis in PTC involves the central compartment first, followed by the ipsilateral lateral compartment and then the contralateral lateral compartment and the mediastinal lymph nodes [9, 10]. However, some patients develop lateral lymph node metastasis (LLNM) in PTC without central lymph node metastasis (CLNM); in these cases, a skip metastasis is noted as positive metastasis to the lateral lymph nodes without the involvement of the central lymph nodes. The frequency of skip metastasis in PTC is approximately 0.6–37.5% [6, 8, 11–24]; however, these estimates come from studies that were limited by low patient numbers and that included primary and recurrent patients in their study population. In addition, although a few studies have assessed the risk factors for skip metastasis in PTC, prospective clinical trials were lacking [25]; preventing determination the intrinsic characteristics of skip metastasis. The aim of the present study was to investigate the incidence, pattern and risk factors of skip metastasis in PTC patients. We retrospectively analyzed clinical data from patients in our hospital who were treated with systematic therapeutic lateral neck dissection.

## Methods

The study was approved by the Institutional Review Board of Zhejiang Cancer Hospital, and formal consent was not required for this research. We retrospectively reviewed the clinical records of 271 patients with PTC and suspicious LLNM diagnosed by pre-operation examinations who underwent total thyroidectomy and central lymph node dissection plus lateral lymph node dissection between January 2008 and December 2011. These patients received their first treatment in the Department of Head and Neck Surgery, Zhejiang Cancer Hospital. All cases were diagnosed with PTC with LLNM by general pathology examination in the department of pathology of our hospital. Patients with other types of thyroid malignancy or with high risk cell types of PTC (e.g., tall cell variant, hobnail variant), with tumors in the isthmus or a family history of PTC were excluded. Patients with a history of neck surgery for other diseases or radiation exposure were also excluded. If the treatment was a palliative surgery, these patients were also excluded.

All patients received a physical examination (PE), ultrasonic examination (US) of the thyroid gland and neck lymph nodes, and neck and thorax computer tomography (CT) with contrast. Fine needle aspiration was not systematically performed in our hospital at the time of this study.The criteria for metastasis requiring US were as follows: round shape (long/short ratio < 2), micro-calcification, cystic change, hyperechogenicity and heterogeneous inner structure [26]. The criteria for CT were as follows: enhancement, heterogeneous, cystic or necrotic change and round shape. The size criteria for both US and CT were based on an upper limit of 15 mm for the nodal diameter of the normal long axis in cases of jugulodigastric and submandibular nodes and 10 mm for all other cervical nodes except for level VI [27].

The initial surgical procedure in our institution was either a bilateral procedure (near-total or total thyroidectomy) or a unilateral procedure (lobectomy) plus bilateral or ipsilateral central compartment dissection. Lateral lymph node dissection was performed if the patient satisfied at least one of the selection criteria; for example, if there was a positive or suspicious pre-operative radiographic finding in the lateral lymph nodes, or if multiple metastatic lateral lymph nodes were identified from the intraoperative frozen biopsy. In this study, all patients underwent total thyroidectomy and therapeutic lateral neck dissection that included levels II to V [28]. Level I was dissected only if there were radiographic, cytopathologic, or intraoperative findings suggestive of metastatic cancer. No patients underwent level I dissection in our study.

The clinical data were retrospectively analyzed with respect to gender, age, tumor size, tumor spread, presence of psammoma bodies, tumor multifocality, extrathyroidal extension (ETE), primary tumor location, CLNM, and LLNM. When multiple lesions were found in the specimen, the largest tumor or the most suspicious dominant nodule was analyzed.

All statistical analyses were two-sided and performed using the Statistical Package for Social Sciences (SPSS, Inc., Chicago, IL, USA). Univariate analyses were performed using the chi-square test and Fisher’s exact test. Data not exhibiting a normal distribution were tested using the Mann-Whitney U test. Variables with *p* < 0.1 in the univariate analyses were included in the multivariate analyses. The multivariate analyses were performed using binary logistic regression analysis to estimate the odds ratios (OR) of individual parameters. The results are presented as ORs with 95% confidence intervals (CI) and *p* values. Some variables were regrouped according to the TNM staging of the tumor according to the American Joint Committee on Cancer (AJCC)/Union for International Cancer Control (UICC) classification system for further binary logistic regression analysis as follows: age ≤ 45 vs. >45 years and tumor location superior vs. elsewhere [29]. For the 6 age categories, 5 dummy variables were introduced, and the first category was selected as the reference category. For the 5 size categories, 4 dummy variables were introduced, and the last category was selected as the reference category. For the 3 capsular invasion categories, 2 dummy variables were introduced, and the first category was selected as the reference category. For the 4 location categories, 3 dummy variables were introduced, and the last category was selected as the reference category. Any *p* value (two-tailed tests) less than 0.05 was considered statistically significant.

## Results

### Demographics of all enrolled patients

In this study, 271 patients were enrolled. There were 64 males and 207 females, representing a male: female ratio of 1:3.23. The age of the patients ranged from 12 to 85 years with a median age of 44.80 years. Tumor diameter ranged from 0.1 to 6.0 cm with a median diameter of 1.5 cm. Among all patients, 47 showed tumor spread in the thyroid gland. A total of 86 patients exhibited ETE, and 31 patients exhibited multifocality in one thyroid lobe. In 75.3% of patients, the primary tumor was located in the upper two-thirds of the lobe (with 107 tumors in the upper part and 97 tumors in the middle) (Table 1). CLNM was present in 191 patients (70.5%), and LLNM was present in 203 patients (74.9%) after post-operative pathological examinations. In this study, a total of 261 patients received total thyroidectomy with bilateral or ipsilateral central compartment dissection plus ipsilateral lateral lymph node dissection, and 10 patients received total thyroidectomy with bilateral central compartment dissection plus bilateral lateral lymph node dissection.

**Table 1:** Clinicopathologicial features of all 271 enrolled patients and univariate analyses results (271 patients)

Characteristics		LLNM negative	LLNM positive	Total	Univariate analysis *p*-value
Gender	Female	55	152	207	0.313
	Male	13	51	64	
Age(year)	≤25	2	19	21	0.087
	25–35	8	41	49	
	35–45	20	64	84	
	45–55	20	49	69	
	55–65	10	19	29	
	>65	8	11	19	
Age(year)	≤45	30	124	154	0.014*
	>45	38	79	117	
Size(cm)	≤0.5	15	10	25	0.000*
	0.5–1	21	44	65	
	1–1.5	12	40	52	
	1.5–2	8	32	40	
	>2	12	77	89	
Tumor Spread	Absent	65	159	224	0.001*
	Present	3	44	47	
Psammoma bodies	Absent	66	195	261	1
	Present	2	8	10	
Multifocality	Single	61	179	240	0.732
	Multi	7	24	31	
ETE	None	58	127	185	0.000*
	ETE	10	76	86	
Location	Upper	8	99	107	0.000*
	Middle	34	63	97	
	Inferior	23	20	43	
	Whole	3	21	24	
Location Upper	Upper	8	99	107	0.000*
	None	60	104	164	
CLNM	Absent	50	30	80	0.000*
	Present	18	173	191	

*symbol for *p* < 0.05; *ETE* extrathyroid extension, *CLNM* central lymph node metastases, *LLNM* lateral lymph node metastasis

In the univariate analyses, LLNM was significantly associated with age, size, tumor spread, ETE, primary tumor location and CLNM (*p* < 0.05), whereas no significant association was found between LLNM and gender, presence of psammoma bodies, multifocality (*p* > 0.05) (Table 1).

The multivariate analysis results are shown in Table 2. The risk factors correctly predicted 86.7% of patients with LLNM. In the multivariate analyses, LLNM was significantly associated with ETE, primary tumor location in the upper part of the thyroid lobe, and a positive finding for CLNM (Table 2).

**Table 2:** Multivariate logistic regression results for LLNM (271 patients)

Predictive factor	*p*	OR	95% C.I. of OR	
			Lower	Upper
ETE(absent versus present)	0.026*	2.756	1.132	6.715
Location(none versus upper)	0.000*	10.471	4.052	27.062
CLNM(absent versus present)	0.000*	20.846	9.505	45.720

*symbol for *p* < 0.05; *ETE* extrathyroid extension, *CLNM* central lymph node metastases, *LLNM* lateral lymph node metastasis

### Prevalence of skip metastasis

In those 203 patients with LLNM, 44 patients showed tumor spread in the thyroid gland, 76 patients exhibited ETE, and 24 patients exhibited multifocality in one thyroid lobe. Additionally, 173 (85.2%) patients presented with CLNM, and 30 (14.8%) patients demonstrated skip metastasis as LLNM without CLNM (Table 3).

**Table 3:** Demographics of 203 patients with lateral compartment metastasis positive (203 patients)

Characteristics		Number of patients	Percentage(%)
Gender	Female	152	74.88
	Male	51	25.12
Age(year)	≤25	19	9.36
	25–35	41	20.20
	35–45	64	31.53
	45–55	49	24.14
	55–65	19	9.36
	>65	11	5.42
Age(year)	≤45	124	61.08
	>45	79	38.92
Size(cm)	≤0.5	10	4.93
	0.5–1	44	21.67
	1–1.5	40	19.70
	1.5–2	32	15.76
	>2	77	37.93
Tumor Spread	Absent	159	78.33
	Present	44	21.67
Psammoma bodies	Absent	195	96.60
	Present	8	3.40
Multifocality	Single	179	88.18
	Multi	24	11.82
ETE	None	127	62.56
	ETE	76	37.44
Location	Upper	99	48.77
	Middle	63	31.03
	Inferior	20	9.85
	Whole	21	10.34
Location Upper	Upper	99	48.77
	None	104	51.23
CLNM	Absent	30	14.78
	Present	173	85.22

*ETE* extrathyroid extension, *CLNM* central lymph node metastases, *LLNM* lateral lymph node metastasis

The 30 patients, 80.0% were female, 50.0% were older than 45 years, and only 10.0% demonstrated tumor spread in the thyroid lobe, with the location of the primary tumor in the upper part of the thyroid lobe in 63.3% of patients. In the univariate analyses, skip metastasis was associated only with tumor size (Table 4).

**Table 4:** Univariate analysis results for skip metastasis (203 patients)

Characteristics		Skip negative	Skip positive	Total	Skip rate(%)	*p*-value
Gender	Female	128	24	152	15.79	0.483
	Male	45	6	51	11.76	
Age(year)	≤25	19	0	19	0	0.065
	25–35	36	5	41	12.2	
	35–45	54	10	64	15.63	
	45–55	41	8	49	16.33	
	55–65	13	6	19	31.58	
	>65	10	1	11	9.09	
Age(year)	≤45	109	15	124	12.1	0.177
	>45	64	15	79	18.99	
Size(cm)	≤0.5	4	6	10	60	0.001*
	0.5–1	39	5	44	11.36	
	1–1.5	35	5	40	12.5	
	1.5–2	26	6	32	18.75	
	>2	69	8	77	10.39	
Tumor Spread	Absent	132	27	159	16.98	0.147
	Present	41	3	44	6.82	
Psammoma bodies	Absent	166	29	195	14.87	1
	Present	7	1	8	12.5	
Multifocality	Single	151	28	179	15.64	0.541
	Multi	22	2	24	8.33	
ETE	None	109	18	127	14.17	0.753
	ETE	64	12	76	15.79	
Location	Upper	80	19	99	19.19	0.387
	Middle	56	7	63	11.11	
	Inferior	18	2	20	10.00	
	Whole	19	2	21	9.52	
Location Upper	Upper	80	19	99	19.19	0.084
	None	93	11	104	10.58	

*symbol for *p* < 0.05; *ETE* extrathyroid extension, *CLNM* central lymph node metastases, *LLNM* lateral lymph node metastasis

For the multivariate analysis, a binary logistic regression was performed, and the results showed that patients with a tumor size ≤0.5 cm had a significantly higher frequency of skip metastasis than did patients with a tumor size larger than 0.5 cm (Table 5).

**Table 5:** Multivariate analysis results for 30 patients with skip metastasis (203 patients)

Predictive factor	*p*	OR	95% C.I. of OR	
			Lower	Upper
Size(>2 cm)	0.010*			
Size(≤0.5 cm)	0.001*	12.937	3.000	55.801
Size(0.5-1 cm)	0.868	1.106	0.338	3.614
Size(1–1.5 cm)	0.731	1.232	0.375	4.046
Size(1.5-2 cm)	0.241	1.990	0.630	6.290

*symbol for *p* < 0.05; Only predictive factors from the multivariate logistic regression model; *OR* odds ratio, *CI* confidence interval

## Discussion

Cervical lymph node metastasis is very common in PTC with an occurrence rate of approximately 18.0–90.0% [2, 3, 30, 31]. Although it remains debated whether lymph node metastasis in patients with PTC is associated with a poor prognosis, there is a consensus that lymph node metastasis at diagnosis can increase the risk of lymph node recurrence, and that re-operation for disease recurrence in the cervical nodes may increase operative complications and medical costs [32, 33].

In this study, the LLNM rate was 74.9% (203/271), which is in agreement with previous reports [2, 3, 31]. However, this rate is much higher than that the 20.0% reported by Patron V, for lateral compartment metastasis in cN0 patients with PTC who had undergone total thyroidectomy and therapeutic lateral neck dissection [34]. In the present study, all patients were suspected cN+, based on radiographic, cytopathologic, intraoperative findings suggestive of metastasis. As a result, therapeutic lateral neck dissection was performed, which might explain the higher LLNM rate observed here than that found in the study by Patron V. Nonetheless, in the present study, 25.1% of the patients did not demonstrate LLNM. All patients received PE, US and contrasted CT examination preoperatively, although fine needle aspiration examination was not systematically performed in our hospital at the time of the study. In recent clinical work, an increasing number of patients have received the fine needle aspiration examination for suspiciously lateral lymph nodes, follow by TG examination if the aspiration results were indeterminate. By performing these evaluation, many unnecessary neck dissections can be avoided.

In previous studies, in which a palpable lateral lymph node was considered a metastatic lymph node, the false-positive rate and false-negative rate were both in the range of 20.0–30.0% [35]. The US/CT combination was superior to US alone in the detection of metastatic lymph nodes at lateral neck levels, which is consistent with other studies [36]. In the present research, if any suspicious radiographic findings were discovered (CT and US), neck dissection was conducted with or without fine needle aspiration examination in our clinic. Of the 41 (15.1%, 41/271) patients who received fine needle aspiration examination of the lateral lymph nodes in our study, 38 patients showed a positive result and demonstrated LLNM in the final pathological examination (18.7%, 38/203). Only 3 patients who received fine needle aspiration examination showed an uncertain result such as dysplasia or a blood component in the non-LLNM group (4.4%, 3/68), which might explain why 25.1% (68/271) of patients did not demonstrate LLNM, making lateral neck dissection unnecessary in these patients. Thus, exhaustive evaluation of the lateral lymph nodes is necessary before the surgery, and aggressive treatment might be avoided especially following fine needle aspiration and TG examination.

In the multivariate analysis, LLNM was significantly associated with ETE (*p* = 0.026, OR = 2.756, 95% CI 1.132–6.715), a primary location in the upper pole of the thyroid lobe (*p* = 0.000, OR = 10.471, 95%CI 4.052–27.062) and CLNM (p = 0.000, OR = 20.846, 95%CI 9.505–45.720) with a positive prediction rate of 86.7%. The significant association of LLNM with primary location. This result might be explained by the hypothesis that the carcinoma cells from the upper region are more likely than those from the lower region to be transported to the lateral lymph nodes by lymphatic flow along the superior thyroid artery [22, 37]. Lymph node metastases arising from primary tumor located in the upper portion of the thyroid lobe in patients with CLNM were more frequent in level II than in the other levels. This suggests that the lymphatic drainage system in the upper portion is different from that in other part of the thyroid lobe [23]. However, several other studies found that location was not significant associated with the pattern of lateral lymph node metastasis [32, 38].

Many previous studies have reported that the dissemination of PTC cells through the lymphatic system occurs in a generally predictable stepwise fashion [7, 8].Lymph node metastasis in PTC involves the central compartment first, followed by the ipsilateral lateral compartment and then the contralateral lateral compartment and the mediastinal lymph nodes [9, 10]. However, some patients have shown LLNM without CLNM, termed skip metastasis, in PTC. The frequency of skip metastasis in PTC is approximately 0.6–37.5% (Table 6) [6, 8, 11–24]. However, the estimates of frequency come from studies were limited by low patient numbers and the inclusion of primary and recurrent patients in their research populations [21]. In this study, a sufficient number of patients (203 patients) underwent simultaneous central and lateral neck dissection for the initial or primary treatment of metastatic PTC. The rate of skip metastasis in the present study was 14.8%, which is range of 0.6% to 37.5% based on previous reports [6, 8, 11–24].

**Table 6:** Summary of previous studies of skip metastasis in PTC (0.6–37.5%)

Authors	Year	LLNM Positive	Skip Positive	Skip rate(%)	Initial Operation	Predictive Factors	Reference
Ducci, Appetecchia et al.	1997	ND	ND	11.1	ND	ND	[11]
Coatesworth and MacLennan	2002	ND	ND	37.5	ND	ND	[12]
Koo, Lim et al.	2010	70	12	17.1	Not for All	NA	[13]
Machens, Holzhausen et al.	2004	66	13	19.7	Not for All	Fewer Positive Lymph Nodes	[8]
Lee, Wang et al.	2007	46	3	6.5	Yes	NA	[14]
Roh, Park et al.	2007	22	3	13.6	Recurrence	NA	[6]
Roh, Kim et al.	2008	52	5	9.6	Yes	NA	[15]
Wada, Masudo et al.	2008	151	17	11.3	Yes	NA	[16]
Chung, Kim et al.	2009	12	3	25	Yes	NA	[17]
Xiao and Gao	2010	64	9	14.1	Yes	NA	[18]
Kim	2012	490	3	0.6	Yes	NA	[19]
Kliseska and Makovac	2012	42	8	19.5	ND	ND	[20]
Lim and Koo	2012	90	17	19	Yes	Lymphovascular Invasion, Extracapsular SpreadAnd fewer Positive Lymph Nodes	[21]
Park, Lee et al.	2012	147	32	21.8	Yes	Single Focus; Locatedupper; Microcarcinoma	[22]
Lee, Shin et al.	2014	131	9	6.8	Yes	Located upper	[23]
Xiang, Xie et al.	2015	44	11	25	Yes	NA	[24]

*NA* None analysis; *ND* No data

We evaluated the predictive factors of the skip metastasis in PTC. In the univariate analyses, the skip rate was significantly associated only with tumor size, and none of the other predictors. In the multivariate analyses, the skip rate was significantly higher in the small tumor size group (especially with tumor size ≤0.5 cm in diameter) than in the other groups (*p* = 0.001, OR = 12.937, 95%CI 3.000–55.801). A tumor with a small size (≤0.5 cm) tends to metastasize to the lateral neck without central compartment metastasis. In the present study, if there was a suspicious thyroid node ≤0.5 cm in the US report (like TI-RADS 4b or 4c), surgery could proceed according to the ATA guideline [39]. As all enrolled patients had suspicious findings for lateral lymph node metastasis preoperative, a total thyroidectomy and neck dissection could proceed in our clinic.

Previous studies have found that skip metastasis is more frequent in less aggressive PTCs such as low-density LLNM and microcarcinomas, which is consistent with our findings [8, 21, 22]. In Lim’s report, the authors proposed three hypotheses for skip metastasis [21]. However, in the present study, less information was collected on the number of positive lymph nodes and the presence of lympho-vascular invasion; analysis of these variables might help identify the predictive factors for skip metastasis.

In the present study, all patients underwent central compartment dissection (lateral or bilateral according to thyroid lesions), and the skip rate (14.8%) was much lower than the classical sequential pattern of the LLNM rate (74.9%). In the 30 patients who demonstrated skip metastasis, 19 patients had a primary tumor located in the upper part of the thyroid lobe. In the univariate and multivariate analyses, the primary tumor location (upper pole) was not significantly different between the skip-positive group and skip-negative group. However, in Lee’s report, 9 patients demonstrated skip metastasis, and all primary tumors were located in the upper part of the thyroid lobe. The authors suggested that these results might reflect the nature of the lymphatic drainage system of the thyroid gland, and Park’s report showed similar results [22, 23]. In Ito’s report, the location of the lesion was significantly associated with the metastasis direction in PTC [22, 37]. The lymphatic drainage system might explain why skip metastasis frequently occurs in patients whose primary tumors are located in the upper portion of the thyroid lobe. Additionally, only a few studies have reported that the location of the PTC is significantly associated with skip metastasis (Table 6). In the present study, the primary tumor location in the upper part of the thyroid lobe was significantly associated with LLNM (*p* = 0.000, OR = 10.471, 95% CI 4.052–27.062), similar to other reports [40]. As shown in Table 3, the skip-positive group, the skip-negative group and the total group showed similar distributions of many clinicpathological features, such as gender, age, and primary tumor location. Therefore, although the primary tumor location may serve as a potential predictive factor for skip metastasis, larger multicenter and long-term follow-up studies are necessary to verify these results.

In addition, our study showed that the skip metastasis was not significantly associated with gender, age, tumor spread, presence of psammoma bodies, capsular invasion, ETE or tumor multifocality. Skip metastasis to the lateral neck in PTC patients occurred more frequently in microcarcinoma patients than in other patients, especially those with a primary tumor size less than 0.5 cm in diameter, which is considered a less aggressive tumor. With only one predictive factor, skip metastasis is difficult to predict and appears to develop in a random fashion.

In the multivariate analysis, tumor intraglandular spread and multifocality were unassociated with LLNM and skip metastasis, similar to some previous reports [19, 21]. PTC frequently presents with multifocal tumors in up to 80% of patients [41]; however, there is currently debate over whether tumor multifocality in PTC represents the intraglandular spread of a single tumor or de novo occurrence of distinct tumors. Jovanovic’s report showed that among papillary thyroid microcarcinomas with multiple tumor foci, 83% had genetic alterations consistent with monoclonal origin based on genome-wide allelotyping and BRAF mutation analysis. The authors suggested that papillary thyroid microcarcinomas were most often mono-clonally derived and that multiple foci developed through the intraglandular spread of an original tumor. The same conclusion was made in Jung’s report; the authors suggest that multifocality might occur during the progression or spread of PTC and represent the intraglandular dissemination of the primary tumor, at least in some cases [42]. It is very difficult to differentiate fully multifocality from intraglandular spread in PTC. The information from the finally result of pathological emanations was employed to classify tumor intraglandular spread and multifocal in the present study.

There are still several potential limitations to this study. This study was limited by its retrospective nature and brief follow-up period. In addition, it investigated only LLNM and skip metastasis. No data were provided regarding other clinicopathological features or long-term follow-up results, such as the numbers of LLNM, other histological subtypes, disease recurrence, postoperative radioiodine studies, thyroglobulin levels, thyroid-stimulating hormone levels, and disease-free survival. We are currently collecting full clinicopathological data and long-term follow-up results for a consecutive report that might be used to improve clinical practice.

## Conclusions

In this study, LLNM was significantly associated with ETE, primary location in the upper pole of the thyroid lobe, and CLNM positive findings; however, skip metastasis was more frequently found in small cancers especially where the primary tumor size was less than 0.5 cm in diameter. In addition, skip metastasis appeared to develop in a random fashion. Thus, although primary tumor location may serve as a predictive factor for skip metastasis, further research involving larger multicenter and long-term follow-up studies is necessary to verify these results.

## Abbreviations

ATA: The American Thyroid Association
CLN: Central lymph node
CLNM: Central lymph node metastases
cN +: Clinical lymph node positive
cN0: Clinical lymph node negative
CT: Computer tomography
ETE: Extrathyroid extension
FNA: Fine needle aspiration cytology
LLN: Lateral lymph node
LLNM: Lateral lymph node metastasis
OR: Odds ratios
PTC: Papillary thyroid carcinoma
SEER: the Surveillance, Epidemiology, and End Results database
SPSS: Statistical Package for Social Sciences
US: Ultrasonic examination or ultrasonography
